# Comparison of Pedicled Adductor Longus and Pedicled Sartorius Flap in Inguinal Reconstruction, a Fresh Cadaver Study

**DOI:** 10.3390/jpm13010143

**Published:** 2023-01-11

**Authors:** Hong Zhang, Zhenfeng Li, Jianmin Li, Binghong Zhu, Qingjia Xu

**Affiliations:** 1 Department of Orthopedics, Qilu Hospital of Shandong University, Jinan 250012, China; 2 Department of Gynecology, The First Affiliated Hospital of Shandong First Medical University and Shandong Provincial Qianfoshan Hospital, Jinan 250014, China

**Keywords:** inguinal region, reconstruction, adductor longus, sartorius, cadaver study

## Abstract

Reconstruction surgeries in the inguinal area are challenging for vascular surgeons, oncologists, orthopedists, and others. The pedicled sartorius flap is the most commonly used flap for reconstruction. The pedicled adductor longus is reported as a new method to reconstruct the inguinal region. The related anatomic study is rare. This work aims to make a comparison of pedicled adductor longus and pedicled sartorius on cadavers for better use. Out of the 12 thighs in the 6 cadavers analyzed, the author compares two surgical methods in terms of surgical details, exposure of vascular and nerve pedicle, flap harvesting, flap transposition and flap volume, etc. Through the course of this study, it is showed that the adductor longus flap had a sizable advantage over the sartorius flap in terms of exposure, harvesting, and flap volume.

## 1. Introduction

Generally speaking, there are two types of procedures carried out in this area: resection procedures and dissection procedures. Local soft tissue defect frequently occurs during oncological surgery around the inguinal region, whether for lymph node dissection or relative tumor resection. Poorly vascularized local soft tissue commonly follows the vascular surgeries around the inguinal area resulting in numerous serious complications. The wound complications following vascular surgery or oncologic resections around the inguinal region have been reported to be up to 40% [1,2,3,4,5,6,7,8,9,10]. Reconstruction of soft-tissue loss around the inguinal region is a tough undertaking, especially for patients who have had oncological resections or who have had complications after vascular surgery. It is more likely that a direct closure without any reconstructions in the inguinal area will lead to a soft tissue defect or dead space, which may cause delayed healing, the wound dehiscence, and a post-operative abscess formation. In order to reduce these complications, surgeons have proposed a variety of methods, including the use of nonabsorbable sutures for skin closure, staples, closed-suction drains, negative pressure wound care, and alternatives of the incision site. Complication rates, however, remain significant. Up to 13–18% of all wound complications have been reported to require reoperation [7,8]. The flaps can provide a well-vascularized coverage to critical structures as well as an essential filling-out of dead space. Thence, many reconstructive methods, including myocutaneous flaps, fasciocutaneous flaps, and muscle flaps, have been published in the previous two decades to reduce inguinal complications. Pedicled muscle flaps described in literatures include sartorius flap (SAF), adductor longus flap (ALF), rectus abdominis flap (RA-M), tensor fascia lata flap (TFL-M), gracilis flap(G-M), and rectus femoris flap (RF-M) [5,11,12,13]. Karl Sörelius and Luigi Schiraldi et al. reviewed the literature of the 538 patients treated for various vascular surgical procedures, and noted that 293 patients (54.5%), out of the total, had their vascular coverage done with a sartorius flap. The sartorius flap is an easy and effective treatment [14]. The adductor longus flap has all the qualities of an ideal donor for a flap. From the authors’ clinical practice, the adductor longus flap is a good alternative for the inguinal region reconstruction [15]. However, the related anatomic study of adductor longus flap, even for the adductor longus, is rare and there is no comparison between the adductor longus flap and the sartorius flap. Hence, the aim of this work is to make a comparison of pedicled adductor longus and pedicled sartorius on cadaver for a better use.

## 2. Materials and Methods

**Figure 1:**
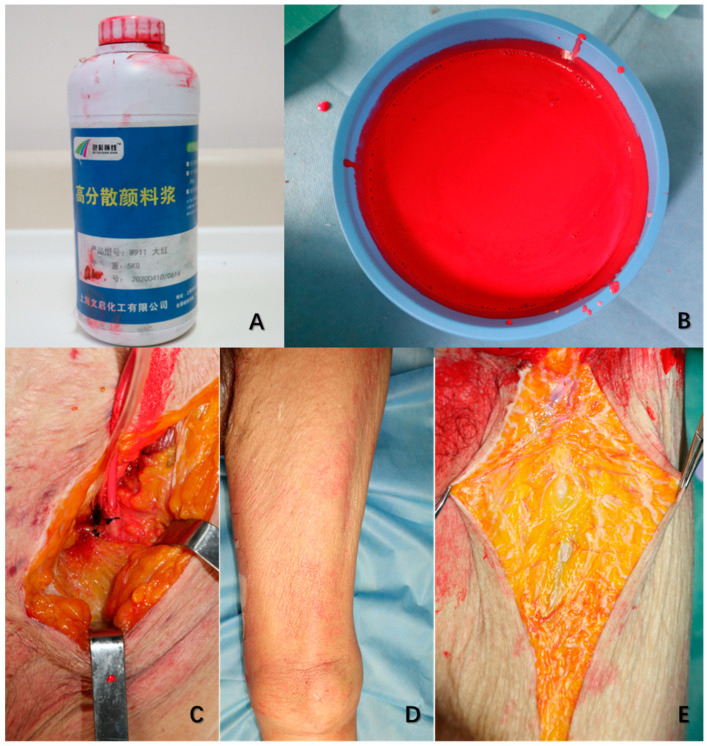
Preparation before the dissection (**A**) is the red polymeric dispersant. (**B**) is the red latex prepared in which the natural latex was mixed thoroughly with the red polymeric dispersant. (**C**) is the perfusion process, and the cadaver is injected with the red latex from B into the femoral artery above the inguinal ligament. (**D**) shows what looks like until the skin of the lower limb flushes pink. (**E**) is just at the beginning of the dissection. The subcutaneous capillaries can be visualized obviously after being perfused with the red latex.

Six fresh cadavers were dissected bilaterally, without evidence of lower limbs’ vascular disease. The mean age of specimens is 63.3 years, ranging from 51 to 75 years. The male/female ratio is 3/1, 4 males and 2 females. The natural latex was mixed thoroughly with the red polymeric dispersant as red latex for perfusion. Before the dissection, the bilateral femoral arteries of all six cadavers were exposed above the inguinal ligament. Then, they were injected with the red latex prepared previously into both femoral arteries until the skin of lower limbs flushed pink (details showed in Figure 1). They were preserved at −20 °C before dissection. All 6 cadavers were simulated with the pedicled adductor longus and the pedicled sartorius flap of bilateral sides at 20 °C room temperature. The perforators and dominant nerves of two different flaps were observed. The author made a comparison of the two procedures in exposure, transportation, fixation, and the volume of the flaps. The cadaver was in supine position, with the leg slightly abducted. An inverted H-shaped incision was used to approach the dissection area. The proximal incision is along with the inguinal ligament, and the distal incision is parallel to the level of the superior border of the patella. Then, an anterior incision above the femoral triangle that originated at the proximal incision and extended distally down to the distal incision was used to expose the dissection area. The plane of dissection was exposed from the superficial to the deep structures between the vastus medialis and sartorius muscles up to the femoral profound artery. The inguinal lymph node with the subcutaneous fat and fascia were removed meticulously to expose the femoral triangle. The saphenous vein was ligated at the saphenofemoral junction and the apex of the femoral triangle after the femoral triangle had been exposed. The femoral canal contents were identified, preserved, and exposed completely. The vessels had a distinctive red color because of the red latex. After that, the author carried out the two flap procedures.

### The Procedures of the Pedicled Adductor Longus Flap and the Pedicled Sartorius Flap

**Figure 2:**
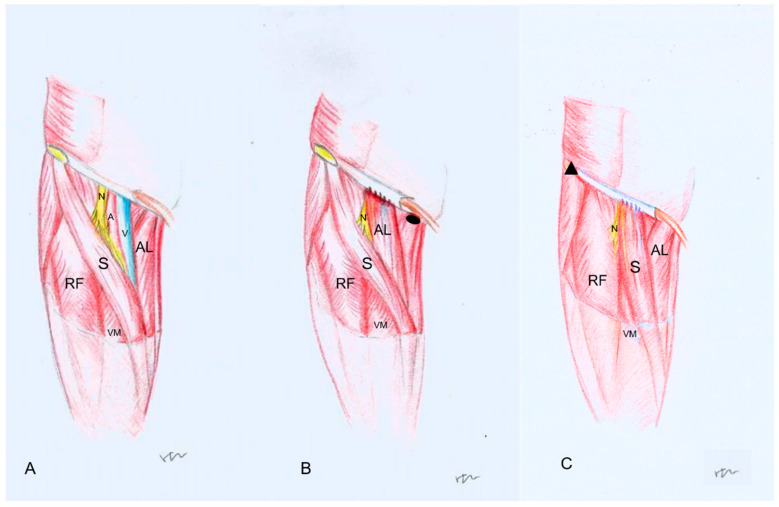
Illustration showing anatomical details of flap reconstruction process: (**A**), after reconstruction with ALF (**B**) and with SAF (**C**) (RF: rectus femoris, AL: adductor longus, S: sartorius, VM: vastus medialis, N: femoral nerve, A: femoral artery, V: femoral vein, black oval: adductor longus attachment of pubis, black triangle: sartorius attachment of superior anterior iliac spine).

**Figure 3:**
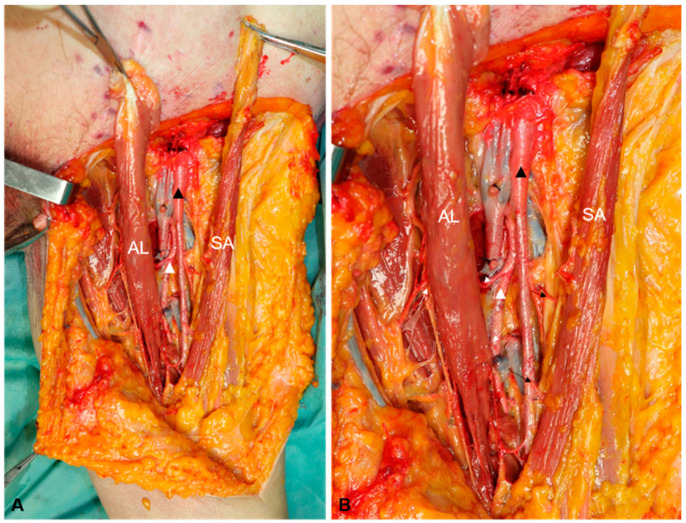
Photos showing two flaps after harvesting (**A**), photos of partial enlargement (**B**) (AL; adductor longus, SA: sartorius, black triangle: femoral artery, white triangle: femoral profound artery, black arrow: muscular perforators of SA).

The proximal insertions of the adductor longus and the sartorius were identified, then they were dissected at the pubis for adductor longus and at the anterior superior iliac spine for sartorius muscle. Both flaps were cautiously peeled to the distal angle of the femoral triangle. To preserve the major muscular perforators and branches, the flap was carefully peeled off. The detached origin was capable of reaching the inguinal ligament after the flap was transferred to cover the critical structures in the femoral triangle, meanwhile the rotation point of the sartorius flap was identified and marked. During the procedures, the terminal branches and dominant nerves of two flaps were preserved and observed. In each case, several photographs of dissected steps were obtained (procedures showed in Figure 2 and dissection showed in Figure 3).

After the two procedures, the two muscle flaps were cut for volume measurement by taking the horizontal line of the rotation point of sartorius muscle flap.

**Figure 4:**
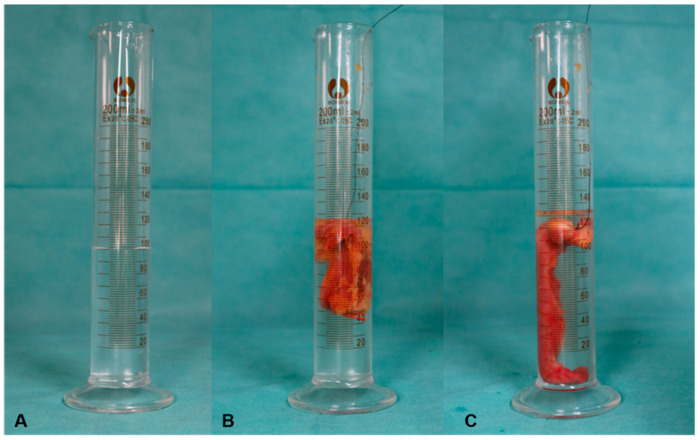
Photos showing how to measure the volume of two flap A volumetric cylinder filled with 100 mL of water before each measurement (**A**), then SAF (**B**), and ALF (**C**), and were submerged in water totally separately reading as 126 mL and 134 mL. The volume of SAF is 26 mL and that of ALF is 34 mL.

Measurement of the volume of the muscle flap: the authors used a 200 mL measuring cylinder filled with 100 mL of water each time and then completely immerged the excised muscle flap in 100 mL water, at which the reading minus 100 mL was the volume of the excised muscle flap. (Figure 4).

The studies involved human cadavers. ‘Clinical and anatomical study of the adductor longus flap in the inguinal reconstruction’ was approved by Medical Ethics Committee of Qilu Hospital of Shandong University with ethics code 2021(073). The registration number of the ethics committee is EC-20200221-1003.

## 3. Results

**Figure 5:**
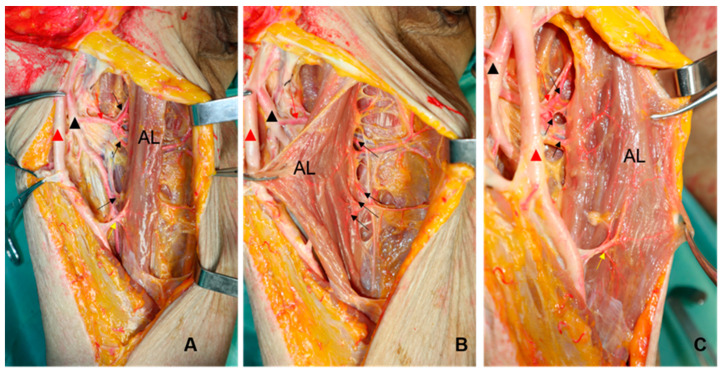
Photographs indicating course of the perforators of adductor longus from the perforating artery of femoral profound artery: (**A**) shows the direct view to the adductor longus, (**B**) with the adductor longus lifted outwards, (**C**) with the adductor longus lifted inwards (red triangle: femoral artery, black triangle: femoral profound artery, AL: adductor longus, red arrow: perforating artery, the branch of femoral profound artery, black arrow: muscular perforators of the adductor longus).

The femoral artery (FA) and the femoral profound artery (FPA) are where the adductor longus’ perforators originate throughout the six cadavers. While those from the FPA run from the center portion of the triangle to below the adductor longus, those from the FA start at the distal part of the femoral triangle above the adductor longus before perforating. The ones from the FPA are thicker in diameter than those from the FA. There are more perforators from the FPA than the FA. The adductor longus’ branches perforate in the middle of the femoral triangle underneath the muscle, whereas the sartorius muscles’ virtually equally perforate at the same height as the sartorius. (Figure 3 and Figure 5) Table 1 presents the overview of all six cadavers including sex, age, cause of death, volume of ALF and SAF on bilateral sides, and the differences and ratio of SAF and ALF on the same side (Table 1).

For the total 12 lower limbs, the mean volume of ALF is 54.7 mL, ranging from 30–130 mL, with a variance of 1147. The mean volume of SAF is 38.2 mL, ranging from 20–89 mL, with a variance of 553.6. The average volume difference between ALF and SAF is 16.5 mL, ranging from 8–41 mL, with a variance of 112.8. The mean ratio of SAF/ALF is 0.70, ranging from 0.63–0.76, with a variance of 0.0025. The mean ratio of left side is 0.72 with a variance of 0.0029, while that of right side is 0.68 with a variance of 0.00167.

## 4. Discussion

The femoral artery, vein, nerve, inguinal node stations, and inguinal canal all meet in the inguinal region as an important anatomical juncture. As a result, the inguinal area is frequently used as the site of surgery for a wide range of interventions from several departments, including surgical lymphadenectomy, diverse oncological resections, and numerous vascular, visceral, and urological surgical procedures. In inguinal region, these could result in soft tissue abnormalities and the exposing of important anatomical structures. Direct closure without any reconstructions for this area is more likely to result in a primary soft tissue defect or dead space, triggering wound dehiscence, delayed healing, and postoperative abscess formation, particularly for vascular surgery. Surgical reconstruction of the inguinal region is difficult due to the anatomical characteristics of inguinal defects between the abdomen and the thigh. Extensive defects with bacterial contamination, non-collapsible dead spaces, lymphatic leaks, and the healing issues with hypovascularization, or ultimately an irradiated area, based on the primary pathology, have been blamed for the poor wound healing in the inguinal region [16,17]. A complication incidence as high as 40% is indicated by the postoperative morbidity associated with inguinal procedures reported in the literature [1].

Regardless of the cause, it is essential to fill in any dead space and create a flap that is well-vascularized and metabolically active for all patients undergoing reconstructive inguinal surgery. Many reconstruction procedures, including myocutaneous flaps, fasciocutaneous flaps, and muscle flaps have been reported during the last two decades [14]. Pedicled muscle flaps reported in the literature are the sartorius flap (SAF), adductor longus flap (ALF), rectus abdominis flap (RAF), tensor fascia lata flap (TFLF), gracilis flap (GF), rectus femoris flap (RFF), and adductor longus flap (ALF) [14,15,17,18,19,20,21,22,23]. Karl Sörelius and Luigi Schiraldi et al. found that in order to lower the risk of infection and reducing the dead spaces, muscle flaps seem to be the best option for the patients following vascular surgery through a system literature review [14]. Among all of the muscle flaps, SAF was the most widely used. The sartorius muscle was first reported as a transposition flap in 1948 to cover exposed femoral arteries post-inguinal lymph node dissection [24]. The sartorius muscle, a type IV (Mathes and Nahai classification) long, thin biarticular muscle, arises from the anterior superior iliac spine (ASIS), runs obliquely, and then inserts onto the medial surface of the proximal tibia [25]. The sartorius muscle is innervated by branches of the femoral nerve. The muscle functions as a thigh flexor, abductor, and external rotator at the hip joint and as a knee flexor at the knee. The circumflex femoral artery and superficial femoral artery supply the proximal one-third of the muscle segmentally, while the superficial femoral vessels feed the middle third. The superficial femoral system, in addition to the branches of the descending genicular artery, vascularizes the distal part of the muscle. When supplied on either the proximal or distal pedicle, studies have shown that more than 80% of muscles survive [26].

The introduction of the perforator flap concept was a result of improvements in microvascular techniques, which revolutionized these reconstruction procedures. The idea of perforator flaps was recently introduced by Wei and Mardini, which created the opportunity to gain novel local reconstructive choices for the inguinal area with decreased donor site morbidity [27,28]. A unique sartorius and adductor fascial flap (SAFF) procedure has recently been published as an alternative to provide autologous tissue covering of the femoral vasculature without requiring the sartorius muscle or the adductor longus to be dissected and rotated [29]. The pedicle adductor longus flap is proven as a new technique for inguinal reconstruction from the authors’ clinical practice. The adductor longus flap has a lot of advantages such as a smaller transportation with better filling-in effect, lower risk of damage to the perforators and dominant nerves, and so on [15]. However, no comparative study of the ALF and SAF procedures has been carried out up until now, especially in cadaver study. In this study, the details of the procedures of ALF and SAF for the reconstruction are observed and compared to each other.

In the course of anatomical simulation operations, the SAF needs to peel more cutaneous fascial flap than the ALF. This could be the reason why Karl Sörelius and Luigi Schiraldi’s summary of the SAF for covering inguinal defects post-vascular surgery, despite showing a lower rate of partial flap necrosis, demonstrated a higher incidence of wound dehiscence attributed to delayed healing of almost 40% [14]. Along the oblique inguinal ligament, the adductor longus is stripped from the pubis to the lateral while the sartorius muscle is from the anterior superior iliac spine to the medial. When the ALF is turned and sutured to the inguinal ligament, there will be some tension, making the ALF need more stiches than the SAF when in fixation to the inguinal ligament, and postoperatively, the patient needs to be placed in a position with the knee and hip flexion as appropriate. Transporting of SAF will not have too much tension and no special requirements for the postoperative position. Through the images of the dissection procedure, it is evident that the ALF requires a smaller shift than the SAF to cover the vascular nerve tracts. (Figure 3).

**Figure 6:**
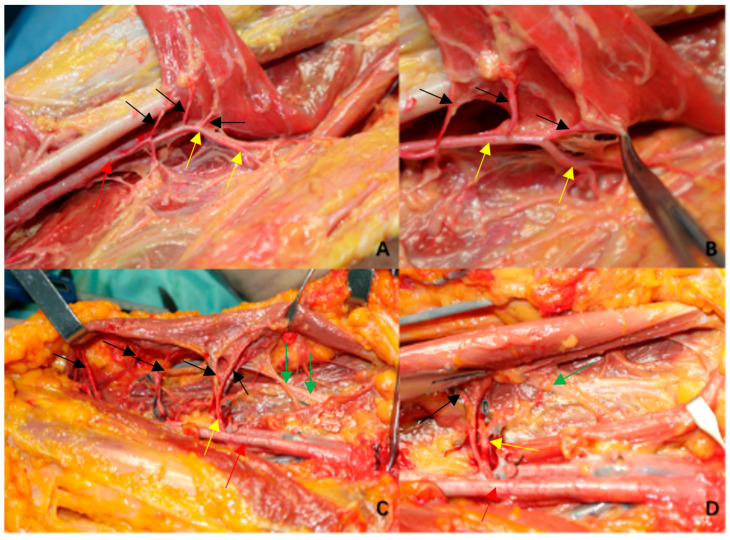
Pictures illustrating how the perforators and branch of ALF go into the muscle (**A**,**B**) are from the No.5 cadaver after the adductor longus lifted from the insertion of the pubic body. (**A**) shows the perforators of adductor longus come from the perforating artery of femoral profound artery. (**B**) expands the perforators of adductor longus in (**A**). (**C**,**D**) are from No.2 cadaver. It is obvious that muscular perforators and dominant nerves of adductor longus flap are beneath the muscle. (Red arrow: femoral profound artery. Yellow arrow: the perforating artery of femoral profound artery. Black arrow: the perforators of adductor longus. Green arrow: the dominant nerves of the adductor longus.).

The position and number of perforators of the profound femoral artery were described in numerous anatomical analyses of the branches of the profound femoral artery, but the scope of the muscular perforators supplying to the particular muscles are not mentioned [30,31]. During our cadaver dissection, it is obvious that muscular perforators and dominant nerves of adductor longus flap are beneath the muscle, the main perforators are from the perforating artery of the femoral profound artery, and the dominate nerves are from the obturator nerve., while the muscular perforators and dominant nerves of sartorius flap are innervated at the same level as the muscle. This anatomical feature makes ALF less likely than SAF to damage perforators and nerves when exposed to the muscle flaps. (Figure 3 and Figure 6).

The thickness of the muscle fibers affects the volumes of SAF and ALF, which are more susceptible to value changes. The fourth specimen’s SAF and ALF values during dissection were much more bilateral than those of the other specimens, and it was obvious from visual observation that the muscle fibers in this specimen were thicker. From Table 1, it is clear that the flap volume measurements for the fourth cadaver were much higher than those of the other groups. Due to the inadequacy of using numbers to depict the volume of the two muscle flaps, we have instead employed the SAF/ALF ratio to express the volume relationship between the two muscle flaps. The results of the autopsy study revealed that the volume ratio of SAF to ALF was close to 0.7, with a variance of 0.0025. As we all know, the femoral triangle (or Scapa’s triangle) is an anatomical region beside the groin, bounded superiorly by the inguinal ligament, medially by the adductor longus, and laterally by the sartorius [32,33,34]. If the adductor longus is separated from the anterior superior iliac spine, the medial bound of the femoral triangle has changed itself as well. The potential cavity of the femoral triangle is inevitably decreased without the ALF filling in. As the ALF is transported laterally to fill out the inguinal area, the dead space of the femoral triangle will be decreased dramatically.

Limitations of the current study include its relatively small sample size, lack of its clinical comparison, and the comparison of postoperative functional evaluation. However, the feasibility and the overall outcome of this cadaver study is reliable.

## 5. Conclusions

The sartorius flap requires more skin and subcutaneous tissue dissection than the adductor longus flap, and there is a higher risk of postoperative skin necrosis and delayed skin healing. The SAF/ALF muscle flap has a volume ratio close to 0.7 and revealed that the SAF is insufficient in cases of larger defects, which instead require the ALF. It demonstrated that the ALFs have a smaller displacement and provide a better fill effect than the sartorius. The smaller displacement of muscle, the less functional impact. The muscular perforators and dominant nerves of the SAF are in the same plane as the sartorius muscle and require extra care to avoid damage before the flap is manipulated. The perforators and nerves of adductor longus flap, on the other hand, are located below the muscle flap, and are usually not affected as long as the muscle and femoral artery are not compromised. SAF is relatively easier to fix than ALF and does not require special postoperative positions to reduce transported muscle tension. SAF can be chosen for lesions that invade medially, while ALF is for lesions that invade laterally.

## Figures and Tables

**Table 1:** The details of the cadavers.

No.	Sex	Age	Cause of Death	Left-Side (mL)				Right-Side (mL)			
				Volume of ALF	Volume of SAF	Volume Difference	Ratio of SAF/ALF	Volume of ALF	Volume of SAF	Volume Difference	Ratio of SAF/ALF
1	M	61	EC ^1^	32	20	12	0.63	40	25	15	0.63
2	F	75	CI ^2^	34	26	8	0.76	38	24	14	0.63
3	F	65	EC ^1^	46	35	11	0.76	49	34	15	0.69
4	M	51	CH ^3^	120	84	36	0.70	130	89	41	0.68
5	M	54	CI ^1^	30	21	9	0.70	53	38	15	0.72
6	M	74	CI ^1^	52	39	13	0.75	32	23	9	0.72

^1^ EC: Esophageal cancer Tables may have a footer. ^2^ CI: Cardiac infarction. ^3^ CH: Cerebral hemorrhage

## Data Availability

Not applicable.

## References

[1] Bandyk D.F. (2008). Vascular surgical site infection: Risk factors and preventive measures. Semin. Vasc. Surg..

[2] Lee E.S., Santilli S.M., Olson M.M., Kuskowski M.A., Lee J.T. (2000). Wound infection after infrainguinal bypass operations: Multivariate analysis of putative risk factors. Surg. Infect..

[3] Nguyen L.L., Brahmanandam S., Bandyk D.F., Belkin M., Clowes A.W., Moneta G.L., Conte M.S. (2007). Female gender and oral anticoagulants are associated with wound complications in lower extremity vein bypass: An analysis of1404 operations for critical limb ischemia. J. Vasc. Surg..

[4] Kent K.C., Bartek S., Kuntz K.M., Anninos E., Skillman J.J. (1996). Prospective study of wound complications in continuous infrainguinal incisions after lower limb arterial reconstruction: Incidence, risk factors, and cost. Surgery.

[5] Brewer M.B., Ochoa C.J., Woo K., Wartman S.M., Nikolian V., Han S., Weaver F.A., Rowe V.L. (2015). Sartorius Muscle Flaps for Vascular Groin Wound Complications. Am. Surg..

[6] Johnson J.A., Cogbill T.H., Strutt P.J., Gundersen A.L. (1988). Wound Complications after Infrainguinal Bypass. Arch. Surg..

[7] Kuy S., Dua A., Desai S., Dua A., Patel B., Tondravi N., Seabrook G.R., Brown K.R., Lewis B.D., Lee C. (2013). Surgical Site Infections after Lower Extremity Revascularization Procedures Involving Groin Incisions. Ann. Vasc. Surg..

[8] Parikh P.P., Rubio G.A., Patel K., Gupta K., Jones K., Rey J., Robinson H. (2018). Transverse versus Longitudinal Incisions for Femoral Artery Exposure in Treating Patients with Peripheral Vascular Disease. Ann. Vasc. Surg..

[9] Audu C.O., Columbo J.A., Sun S.J., Perri J.L., Goodney P.P., Stone D.H., Nolan B.W., Suckow B.D. (2019). Variation in timing and type of groin wound complications highlights the need for uniform reporting standards. J. Vasc. Surg..

[10] Fischer J.P., Nelson J.A., Mirzabeigi M.N., Wang G.J., Foley P.J., Wu L.C., Woo E.Y., Kanchwala S. (2012). Prophylactic muscle flaps in vascular surgery. J. Vasc. Surg..

[11] Shermak M.A., Yee K., Wong L., Jones C.E., Wong J. (2005). Surgical Management of Groin Lymphatic Complications after Arterial Bypass Surgery. Plast. Reconstr. Surg..

[12] Illig K.A., Alkon J.E., Smith A., Rhodes J.M., Keefer A., Doyle A., Serletti J., Shortell C.K., Davies M.G., Green R.M. (2004). Rotational Muscle Flap Closure for Acute Groin Wound Infections Following Vascular Surgery. Ann. Vasc. Surg..

[13] Herrera F.A., Kohanzadeh S., Nasseri Y., Kansal N., Owens E.L., Bodor R. (2009). Management of Vascular Graft Infections with Soft Tissue Flap Coverage: Improving Limb Salvage Rates–A Veterans Affairs Experience. Am. Surg..

[14] Sörelius K., Schiraldi L., Giordano S., Oranges C.M., Raffoul W., Di Summa P.G. (2018). Reconstructive Surgery of Inguinal Defects: A Systematic Literature Review of Surgical Etiology and Reconstructive Technique. In Vivo.

[15] Zhang H., Li Z., Li J., Zhu L., Ibrahim Y. (2021). The Pedicled Flap of Adductor Longus, a New Technique for Inguinal Reconstruction. Front. Surg..

[16] Murthy V., Gopinath K.S. (2012). Reconstruction of groin defects following radical inguinal lymphadenectomy: An evidence based review. Indian J. Surg. Oncol..

[17] LoGiudice J.A., Haberman K., Sanger J.R. (2014). The anterolateral thigh flap for groin and lower abdominal defects: A better alternative to the rectus abdominis flap. Plast. Reconstr. Surg..

[18] Nahai F., Hill L.H., Hester R.T. (1979). Experiences with the Tensor Fascia Lata Flap. Plast. Reconstr. Surg..

[19] Ali A.T., Rueda M., Desikan S., Moursi M.M., An R., Spencer H., Rueda S., Eidt J.F. (2016). Outcomes after retroflexed gracilis muscle flap for vascular infections in the groin. J. Vasc. Surg..

[20] Armstrong P.A., Back M.R., Bandyk D.F., Johnson B.L., Shames M.L. (2007). Selective application of sartorius muscle flaps and aggressive staged surgical debridement can influence long-term outcomes of complex prosthetic graft infections. J. Vasc. Surg..

[21] Chateau F., Duisit J., Lengel B., Vanwijck R. (2010). Techniques for coverage of infected vascular grafts in the groin. Acta Chir. Belg..

[22] De Santis F., Chaves Brait C.M., Caravelli G., Pompei S., Di Cintio V. (2013). Salvage of infected vascular graft via ‘perivascular venous banding’ technique coupled with rectus abdominis myocutaneous muscle flap transposition. Vascular.

[23] Ducic I., Dayan J.H., Attinger C.E., Curry P. (2008). Complex perineal and groin wound reconstruction using the extended dissection technique of the gracilis flap. Plast. Reconstr. Surg..

[24] Baronofsky I.D. (1948). Technique of inguinal node dissection. Surgery..

[25] Buckland A., Pan W.R., Dhar S., Edwards G., Rozen W.M., Ashton M.W., Taylor G.I. (2009). Neurovascular Anatomy of Sartorius Muscle Flaps: Implications for Local Transposition and Facial Reanimation. Plast. Reconstr. Surg..

[26] Manjunath K.N., Venkatesh M.S., Shivaprasad A. (2018). Distal major pedicle of sartorius muscle flap: Anatomical study and its clinical implications. Indian J. Plast. Surg..

[27] Mardini S., Tsai F.-C., Wei F.-C. (2003). The thigh as a model for free style free flaps. Clin. Plast. Surg..

[28] Wei F.-C., Mardini S. (2004). Free-Style Free Flaps. Plast. Reconstr. Surg..

[29] Lattimore C.M., Meneveau M.O., Marsh K.M., Shada A.L., Slingluff C.L., Dengel L.T. (2022). A Novel Fascial Flap Technique After Inguinal Complete Lymph Node Dissection for Melanoma. J. Surg. Res..

[30] Siddharth P., Smith N.L., Mason R.A., Giron F. (1985). Variational anatomy of the deep femoral artery. Anat. Rec..

[31] Kalinin R.E., Suchkov I.A., Klimentova A., Shanaev I.N. (2021). Clinical anatomy of deep femoral vessels in the area of femoral triangle. Angiol. Vasc. Surg..

[32] Garg K. (2010). Front of the thigh. Chaurasia BD. BD Chaurasia’s Human Anatomy (Regional and Applied Dissection and Clinical).

[33] Williams P.L. (1995). Gray’s Anatomy.

[34] Benninghoff (1985). Makroskopische und mikroskopische Anatomie des Menschen.

